# L-Dopa-induced changes in aperiodic bursts dynamics relate to individual clinical improvement in Parkinson’s disease

**DOI:** 10.1038/s41531-025-01024-w

**Published:** 2025-06-10

**Authors:** Hasnae Agouram, Matteo Neri, Marianna Angiolelli, Damien Depannemaecker, Jyotika Bahuguna, Antoine Schwey, Jean Régis, Romain Carron, Nicole Malfait, Alexandre Eusebio, Emmanuel Daucé, Pierpaolo Sorrentino

**Affiliations:** 1 Aix Marseille Univ, CNRS, INT, Institut de Neurosciences de la Timone, Marseille, France; 2 Ecole Centrale Méditerranée, Marseille, France; 3 Aix-Marseille Univ, INSERM, INS, Institut de Neurosciences des Systèmes, Marseille, France; 4 Unit of Nonlinear Physics and Mathematical Models, Department of Engineering, Campus Bio-Medico University of Rome, Rome, Italy; 5 Laboratoire de Neurosciences Cognitives et Adaptatives, Université de Strasbourg, Strasbourg, France; 6 Aix Marseille Univ, UMR INSERM 1106, Dept of Functional Neurosurgery, Marseille, France; 7 Medico-surgical Unit Epileptology, Functional and Stereotactic Neurosurgery, Timone University Hospital, Marseille, France; 8 APHM, Hôpitaux Universitaires de Marseille, Hôpital de la Timone, Department of Neurology and Movement Disorders, Marseille, France

**Keywords:** Parkinson's disease, Parkinson's disease, Parkinson's disease

## Abstract

Parkinson’s disease (PD) is a neurodegenerative disease characterized by severe motor symptoms, transiently alleviated by medication (e.g. levodopa), and widespread brain activity alterations that remain poorly understood at a large scale level. To address this issue, we used resting-state STN-DBS and motor EEG data from 11 PD patients before and after levodopa treatment. Neuronal avalanches, i.e., brief, widespread bursts of activities, were detected and compared across the two conditions. Interestingly, we noted shorter and smaller avalanches in the OFF-condition and fewer, longer, and larger avalanches in the ON-condition. We then computed the avalanche transition matrices to track the contact-wise patterns of avalanche spread. We found a significantly higher probability of avalanche spread within and between the STN and motor cortex in the ON-condition. Furthermore, increased propagation of avalanches correlated with clinical improvement. Our study identifies potential biomarkers for electrophysiological changes in PD through cross-modality assessment of aperiodic activities.

## Introduction

Parkinson’s disease (PD) is a chronic and progressive neurological disorder that affects movement, with key motor symptoms including tremor, rigidity, bradykinesia, and postural instability^1,2^. Additionally, the clinical picture also encompasses several non-motor symptoms such as depression, sleep disturbances, and dementia^3,4^. The pathological hallmark of PD involves the destruction of dopaminergic neurons in the pars compacta of the substantia nigra^3,5,6^. Previous work showed that substantia nigra malfunction affects the entire brain, which is mirrored by widespread alterations in neuronal activities in PD patients^7–11^. In particular, interactions within the basal ganglia-thalamocortical circuit have been central in characterizing and studying the mechanisms underpinning this disease^12^. To investigate these, we leveraged a unique dataset with simultaneous bilateral recordings: EEG in the motor cortex and deep stimulation electrodes in the subthalamic nuclei (STN). Such an approach may prove beneficial in bridging the gap between local pathological changes (e.g. within the cortical-BG-thalamic loop) and the corresponding clinical manifestations in PD (by elucidating how local alterations reverberate across the whole brain).

Classically, large-scale interactions in PD have been understood in terms of periodic oscillations and their modulation (i.e. beta bursts)^10,13–17^. More recently, a new perspective has focused on the scale-free properties of large-scale activities^18,19^. Aperiodic, spontaneous bursts spreading across a range of spatial and temporal scales (“neuronal avalanches”) have been consistently observed across different imaging modalities and spatio-temporal scales^20–22^. Neuronal avalanches, manifesting as bursts of activations spreading across multiple brain signals, characterize the large-scale interactions across multiple brain regions, and constitute a marker of physiological states, for example allowing the characterization of conditions such as sleep and resting wakefulness^20,23^, or speech and music listening^24^. Furthermore, avalanche dynamics were shown to be altered in PD patients, and proportionally to clinical impairment^25^.

While avalanches have been classically analyzed in terms of their global statistical properties (e.g. the distribution of their sizes, durations, flexibility, etc.), a more recent approach focused on the topography and topology of such bursts across the brain. To this end, the avalanche transition matrices (ATMs) have been developed to track from where to where avalanches spread on average across the brain. As a result, it became evident that the spatial and temporal propagation patterns differ between healthy state and pathology^25–29^.

In the present work, we deploy for the first time the analysis of avalanches in a cross-modality setting, under the hypothesis that the administration of levodopa changes the spreading of aperiodic bursts of activities between the motor cortex and the subthalamic nuclei. To achieve this, we used resting-state data from 11 PD patients (partly overlapping with the dataset utilized in ref. ^30^), recorded in two conditions: ON-levodopa and OFF-levodopa medication. We hypothesize that the changes induced by levodopa on large-scale activities can be understood in terms of changes in the topology of scale-free perturbations, manifesting as different propagation patterns and, more generally, different statistical properties of large-scale perturbations. As such, avalanches would present different statistical features and propagate differently according to the patient’s medication state which, in turn, would be related to clinical symptoms. Hence, the ATMs might be seen as a potential marker to track both the medication effects and motor improvement at the individual level.

## Results

### Levodopa decreases the avalanche rate but increases the avalanche size

**Fig. 1:**
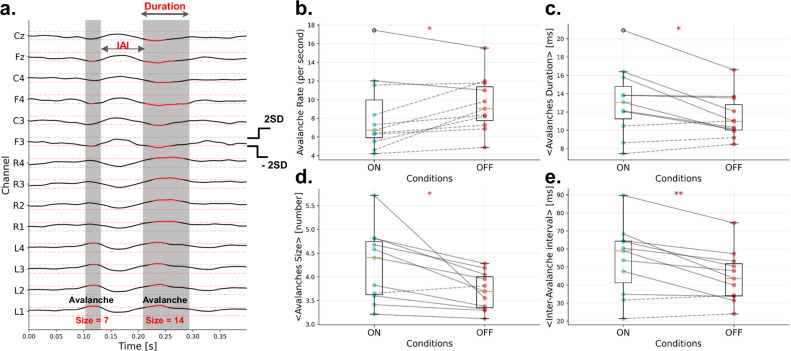
Group-level analysis of avalanche features. A neuronal avalanche is defined as a continuous sequence of signal excursions beyond the threshold (red thick line) of one or more channels, **a** Avalanche features illustration (adapted from ref. ^23^). The avalanche duration is defined as the total length of an avalanche, while the avalanche size corresponds to the number of recording sites recruited during the avalanche. An avalanche is preceded and followed by intervals during which signals in all channels fall below the threshold (i.e. inter-avalanche intervals). **b** Avalanche Rate Comparison: Comparison of avalanche rate per second between ON- and OFF-levodopa conditions at the group level. Dashed lines indicate higher values in the OFF condition compared to ON, while solid lines represent higher values in the ON condition compared to OFF. **c** Avalanche Duration: Group-level comparison of average avalanche duration (ms) for each patient. **d** Avalanche Size: Group-level comparison of average avalanche size (# of channels) for each patient. **e** Inter-Avalanche Interval: Group-level comparison of average inter-avalanche interval (ms) for each patient. For (**b**–**e**), we tested the significance of the differences in the distributions between ON-levodopa and OFF-levodopa conditions using the paired t-test, resulting in the following effect sizes, measured as Cohen’s d (denoted by d), and *p*-values (denoted by *p*): avalanche rate (*d* = 0.66, *p* = 0.033), duration (*d* = 0.77, *p* = 0.028), size (*d* = 0.90, *p* = 0.013), and inter-avalanche interval (*d* = 1.06, *p* = 0.0054). The significance level are indicated as ***p < 0.001, **p < 0.01, *p < 0.05, ns non-significant, *p* refers to *p*-value.

Neuronal avalanches have been estimated (after signal preprocessing, see Methods) by binarizing the z-scored activities. Each brain signal, including EEG and LFP, was independently z-scored across time to identify salient events in neural dynamics that exceeded a threshold of 2 standard deviations (|z| = 2). When a brain signal has a z-score above threshold it is set to 1 (*active*); in all the other time points it is set to 0 (*inactive*). A neuronal avalanche starts when at least one channel becomes active, and ends when all channels become inactive. Neuronal avalanches are characterized by their size, *s*, defined as the number of channels recruited during the avalanche; their duration, and the inter-avalanche interval (IAI), defined as the time interval between two consecutive avalanches (Fig. 1a).

To characterize the two conditions (i.e. ON vs OFF), we first compared the number of avalanches at the subject level (Supplementary Fig. 1a). We found that avalanches occur more frequently in the OFF-levodopa condition as compared to the ON-levodopa condition in nine patients out of eleven. More specifically, we compared the total number of avalanches per 4-second segment. While we reached significance only for four patients out of eleven (Mann–Whitney test), there were more avalanches in the OFF-state as compared to the ON-state in all patients except for two. The group-level analysis (Fig. 1b), based on a paired t-test, confirmed a significant difference in the avalanche rate (*p* = 0.033; *p* refers to *p*-value) with an effect size of 0.66, measured as Cohen’s d. For more details about subject-level analysis, we compared the distributions of the number of avalanches across 4-s segments for each patient in the ON-state and the OFF-state (Supplementary Fig. 2).

Then, we compared the duration (Supplementary Fig. 1b), size (Supplementary Fig. 1c), and inter-avalanche interval (IAI) of avalanches (Supplementary Fig. 1d) at the subject level and we observed a common trend across the majority of the patients: the average size, duration, and inter-avalanche interval (IAI) are larger in the ON-levodopa condition than in the OFF-levodopa condition. While we do not reach statistical significance for all patients utilizing the two-sample Kolmogorov–Smirnov (K-S) test (the reason for choosing the K-S test can be found in the *Methods* section), the group-level analysis (Fig. 1c–e) confirmed a larger size, duration, and inter-avalanche interval in the ON-levodopa condition, with significant paired t-test results: size (*d* = 0.90, *p* = 0.013), duration (*d* = 0.77, *p* = 0.028), and inter-avalanche interval (*d* = 1.06, *p* = 0.0054). Effect size is reported as Cohen’s d, denoted by *d*. Overall, our first analysis indicates a higher number of avalanches in the OFF-levodopa state, but larger avalanche size, larger avalanche duration, and longer inter-avalanche intervals in the ON-levodopa state. In conclusion, in the ON-levodopa state, we observe a reduced number of avalanches, yet they exhibit longer durations, greater sizes, and longer intervals between them. For additional details, please refer to Supplementary Figs. 3–5 for visualizations of the comparison of the cumulative distribution functions of avalanche sizes, avalanche durations, and inter-avalanche intervals for each patient in the ON-state and OFF-state, along with the results of the Kolmogorov–Smirnov (K-S) test.

### Levodopa facilitates the spreading of avalanches

**Fig. 2:**
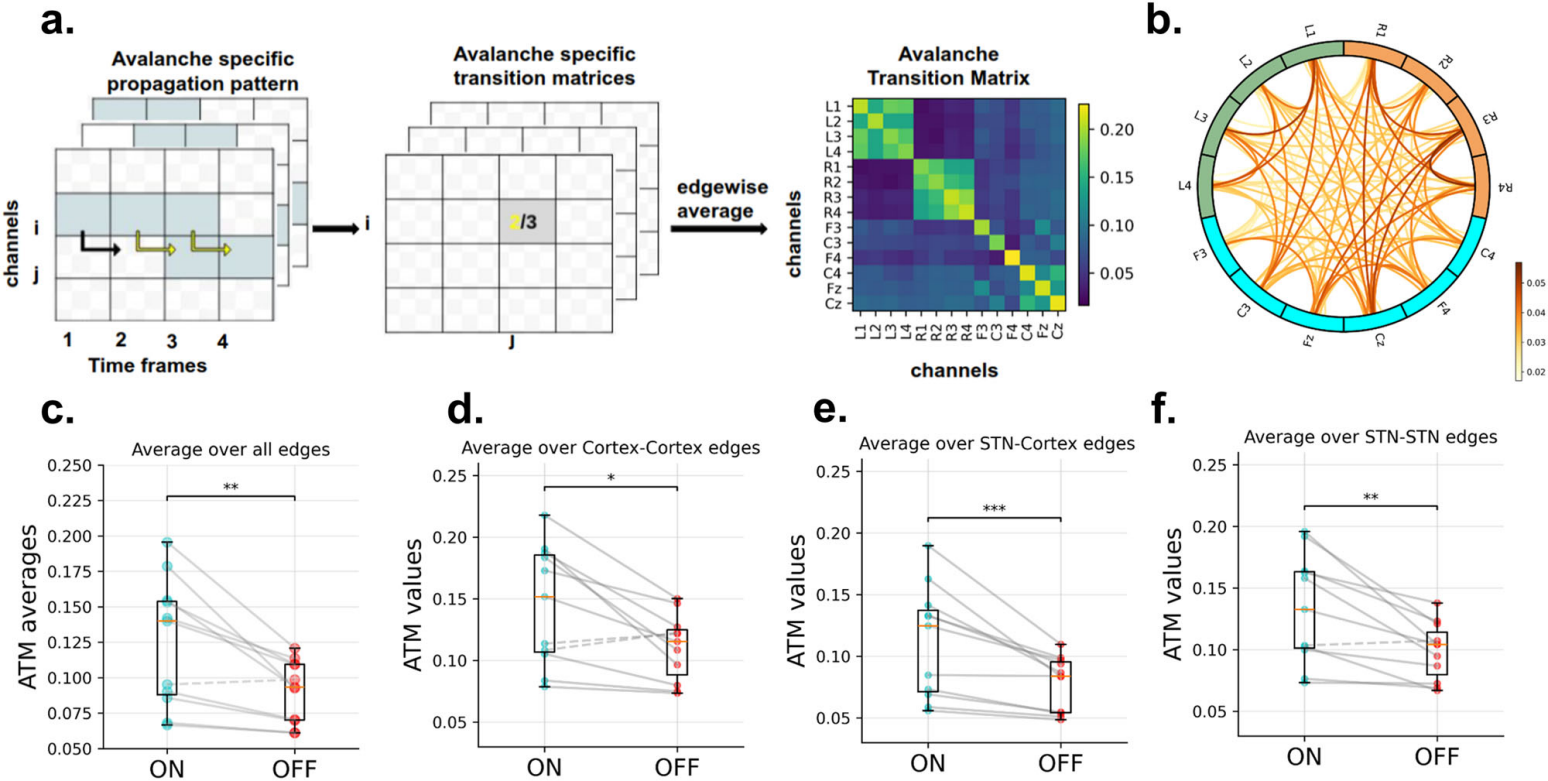
Avalanche Transition Matrix (ATM) in ON-levodopa and OFF-levodopa conditions. **a** Avalanche Transition Matrix pipeline. The central and left panels are adapted from ref. ^57^. The light blue squares signify that channel i exceeded the threshold three times during the avalanche. Channel j was active following the activation of channel i in just two out of the three cases considered (as shown by the yellow arrows), yielding a probability of 2/3. An individual’s avalanche transition matrix is obtained by averaging over avalanche-specific transition matrices. Right panel: ATMs are structured by channels in rows and columns; the resulting shape being (channels,channels) = (14,14). The color bar shows transition probability values. **b** Average edge difference across patients (circular graph representation) the edge color reports the average difference between the ATMs in ON and OFF-levodopa conditions, across all patients. The left LFP contacts shown in green, right LFP contacts in orange and EEG channels in blue. Regarding the edges, the intensity of the color is proportional to the strength of the difference. In (**c**) Group level ATM average comparison in the ON-levodopa and OFF-levodopa conditions. Each dot represents one patient. The significance of the differences was assessed with the Wilcoxon signed-rank test, resulting in a Wilcoxon effect size of 0.85 and *p*-value of 0.0019. **d** Group level Cortico-Cortical edges average comparison, **e** Group level STN-Cortical edges average comparison, **f** Group level STN-STN edges average comparison. Dashed lines indicate higher values in the OFF condition compared to ON, while solid lines represent higher values in the ON condition compared to OFF. The statistical significance of the differences was tested with the Wilcoxon signed-rank test, resulting in the following Wilcoxon effect sizes (denoted by *r*) and *p*-values (denoted by *p*): cortex-cortex edges (*r* = 0.72, *p* = 0.013), STN-cortex edges (*r* = 0.88, *p* = 0.00097), and STN-STN edges (*r* = 0.83, *p* = 0.0029). The significance level are indicated as ***p < 0.001, **p < 0.01, *p < 0.05, ns non-significant, *p* refers to *p*-value.

To characterize how avalanches spread throughout the brain, we computed the avalanche transition matrices^31^. An avalanche-specific transition matrix was calculated, where element *(i, j)* represents the probability that channel *j* was active at time *t* + *1*, given that channel *i* was active at time *t*. For each patient, we obtained an average transition matrix (ATM) (i.e. averaging edge-wise over all avalanches) and then symmetrized it (Fig. 2a). The matrices were symmetrized to help the interpretability of the results and to obtain more reliable estimates. Hence, we obtained two symmetric ATMs for each patient, one for the ON condition and one for the OFF condition, see Methods.

In other words, ATMs contain values between 0 and 1, which are average probabilities of two nodes to be successively activated by an avalanche during the recording. As observed in Supplementary Fig. 6a, b, by averaging all the ATMs in each condition, the probability is higher within each region (i.e. within the left STN, the right STN and the Cortex) than across the areas. However, a clear difference is also visible when we look at the inter-regional links. The darker color in the ON condition indicates that across-region avalanches are more frequent. The average difference between the ATMs (that is, ON-levodopa minus OFF-levodopa) is positive for all the edges across patients (Fig. 2b). This difference is widespread across the regions, and even more pronounced in the right hemisphere. On average, the probability of propagation of avalanches is higher in the ON-levodopa than the OFF-levodopa state, across all pairs of recording sites.

This is confirmed by a statistical analysis. At the group level, the average of ATMs across all edges is significantly higher in the ON-levodopa than in the OFF-levodopa condition (Fig. 2c, Wilcoxon signed-rank test; mean_ON = 0.12, std_ON = 0.042, mean_OFF = 0.090, std_OFF = 0.020, *r* = 0.85, *p* = 0.0019, *r* refers to the Wilcoxon effect size). This is true for all the patients of the group except for one. To assess regional effects, we classified the edges into three groups: *STN-STN*, edges connecting two channels both located in the STN (recorded using deep brain stimulation electrodes), *cortex-cortex*, edges connecting two channels located both in the motor cortex (recorded using EEG), and *STN-cortex*, that is edges with one channel located in the STN and the other located in the cortex. We found that the average of each of these was significantly higher during the ON-levodopa than in the OFF-levodopa conditions at the group level (Fig. 2d–f, Wilcoxon signed-rank test; cortex-cortex: mean_ON = 0.14, std_ON = 0.046, mean_OFF = 0.11, std_OFF = 0.025, *r* = 0.72, *p* = 0.013; STN-cortex: mean_ON = 0.11, std_ON = 0.043, mean_OFF = 0.078, std_OFF = 0.021, *r* = 0.88, *p* = 0.00097; STN-STN: mean_ON = 0.13, std_ON = 0.042, mean_OFF = 0.098, std_OFF = 0.022, *r* = 0.83, *p* = 0.0029, *r* refers to the Wilcoxon effect size). Furthermore, consistent with expectations regarding avalanche propagation within the STN compared to STNs from different hemispheres, we found that avalanche propagation is higher within the STN than between STNs of different hemispheres (Supplementary Fig. 7; Wilcoxon signed-rank test; within the left STN (mean_ON = 0.19, std_ON = 0.048, mean_OFF = 0.15, std_OFF = 0.037, *r* = 0.75, *p* = 0.0097); between the left and right STNs (mean_ON = 0.066, std_ON = 0.039, mean_OFF = 0.040, std_OFF = 0.016, *r* = 0.88, *p* = 0.00097); and within the right STN (mean_ON = 0.20, std_ON = 0.053, mean_OFF = 0.16, std_OFF = 0.042_,_ *r* = 0.77, *p* = 0.0068)). *r* refers to the Wilcoxon effect size.

Those results confirm the effects of levodopa medication on avalanche dynamics in the interaction between the STN and the motor cortex, reaching a statistically significant difference (*p* < 0.001) at the level of the STN-cortex edges. This can be interpreted as a facilitated propagation of neuronal avalanches under levodopa medication, this effect being more manifest across regions when considering the subcortical contacts together with the EEG channels. Furthermore, this analysis supports our findings indicating that these are not dependent on modality.

### Increased propagation of avalanches between STN and cortex correlates with clinical improvement

**Fig. 3:**
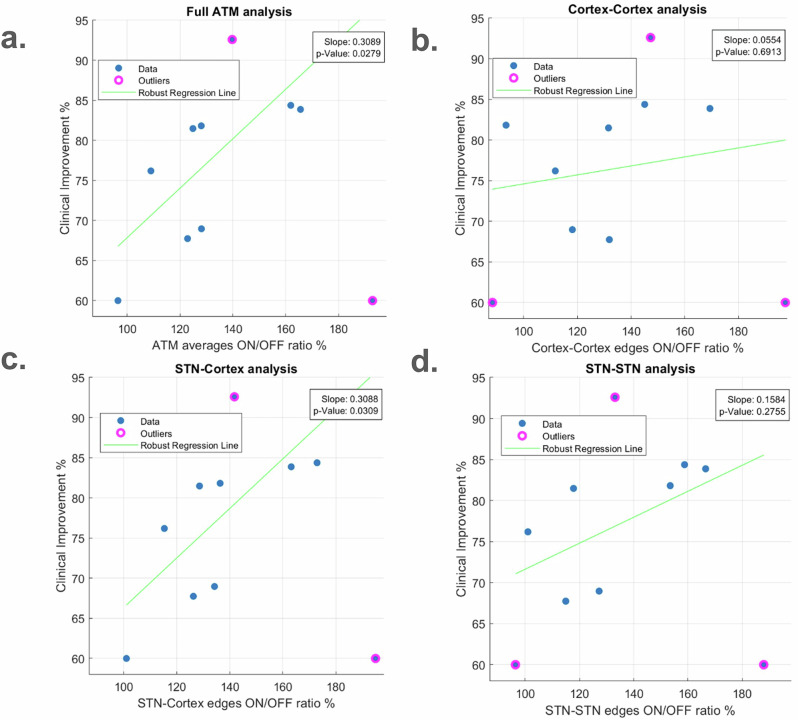
Group level clinical improvement correlation analysis. **a** ATM averages ON/OFF ratio vs. clinical improvement. Robust linear regression. Each patient is represented as a blue dot, except for outliers with a pink circle. The regression coefficient was 0.3089, the *p*-value was 0.0279, and the t-statistic was 2.6810. **b** cortex-cortex edges ON/OFF ratio vs. clinical improvement. **c** STN-cortex edges ON/OFF ratio vs. clinical improvement. **d** STN-STN edges ON/OFF ratio vs. clinical improvement. Robust linear regression. Each patient is represented as a blue dot, except for the outliers with a pink circle. The regression coefficients (denoted by *r*), *p*-values (denoted by *p*), and t-statistics were as follows: cortex-cortex edges (*r* = 0.0554, *p* = 0.6913, t-statistic = 0.4118); STN-cortex edges (*r* = 0.3088, *p* = 0.0309, t-statistic = 2.6156); and STN-STN edges (*r* = 0.1584, *p* = 0.2755, t-statistic = 1.1704).

Furthermore, UPDRS III scores (*Unified Parkinson’s Disease Rating Scale part III*) were assessed before and after levodopa administration. We thus defined a clinical improvement ratio (see Methods), reflecting a percentage of clinical improvement with respect to the baseline score. Then, we assessed the correlation between the clinical improvement and the ratio of the mean ATMs in the ON versus OFF states using robust linear regression. This analysis was conducted on ten out of eleven patients, excluding patient number eight due to unavailable clinical data (see Methods). A significant positive correlation was found (*r* = 0.3089, *p*-value = 0.0279, t-statistic = 2.6810), see Fig. 3a, indicating a possible relation between avalanche spread pattern and clinical improvement : as the overall propagation of avalanches increases, the mean clinical improvement after levodopa administration also tends to increase.

We also observed similar results when assessing the relationship of clinical improvement with the ratio between the averages over specific types of edges in the ATM (i.e. cortex-cortex, STN-cortex, and STN-STN) in the ON and OFF states. Our findings indicated a positive correlation for the three types of edges, but it only reached significance for the STN-cortex edges (for cortex-cortex edges, the values were (*r* = 0.0554, *p*-value = 0.6913, t-statistic = 0.4118); for STN-cortex edges (*r* = 0.3088, *p*-value = 0.0309, t-statistic = 2.6156); and for STN-STN edges (*r* = 0.1584, *p*-value = 0.2755, t-statistic = 1.1704)), see Fig. 3b–d. Therefore, clinical improvement appears to relate more specifically to the large-scale coordination of activities, in particular between the STN and the motor cortex.

Furthermore, we studied the clinical correlation with total number of avalanches, average duration, average size, and average inter-avalanche interval; however, no significant relationships were identified (see Supplementary Fig. 8).

**Fig. 4:**
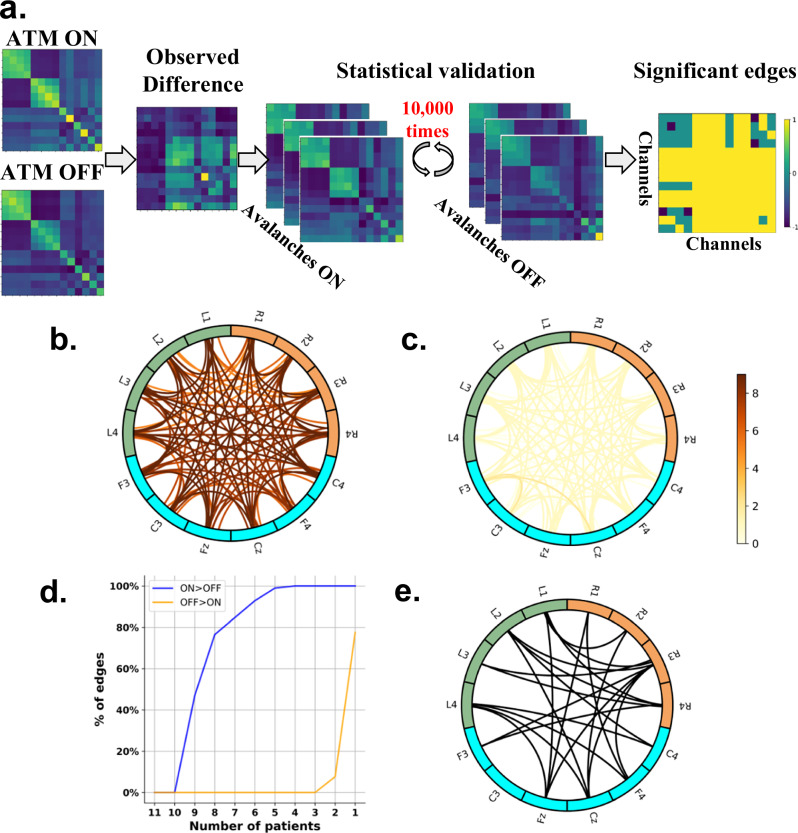
Edge-wise analysis. **a** Statistical pipeline used to identify the edges that exhibit significant differences between the ON-levodopa and OFF-levodopa conditions adapted from ref. ^58^. The ATM_ON_ (ATM in ON-levodopa state) was subtracted, edge-wise, from the ATM_OFF_ (ATM in OFF-levodopa state). The significance is assessed by a permutation test, through iteratively randomizing the labels of avalanche-specific transition matrices (i.e., ON-levodopa medication and OFF-levodopa medication) 10,000 times, before averaging and computing the differences. The *p*-values are calculated as the proportion of random differences (separately for ATM_ON_ - ATM_OFF_ and ATM_OFF_ - ATM_ON_) greater than the observed difference for each patient. *P*-values are considered significant when <0.05 after Benjamini–Hochberg (BH) correction for multiple comparisons. **a** right panel: significance result for one patient; in this representation, the color code corresponds to the three possible results either ON > OFF (value = 1), OFF > ON (value = −1), or non-significant edges (value = 0). **b** Cumulative ON > OFF edge consistency. The color code reflects the number of significant occurrences ON > OFF across patients, with 9 being the max consistency observed. **c** Cumulative OFF > ON edge consistency. The color code is similar to panel (**b**). **d** Percentage of edges that are significant for at least *n* patients, 1 ≤ *n* ≤ 11 in both cases: the ON-levodopa greater than OFF-levodopa state, and the OFF-levodopa greater than ON-levodopa state. **e** Edge-wise group-level analysis. The statistical significance of the differences was tested with the Wilcoxon signed-rank test; significant edges (*p*-values < 0.01) are shown after applying the BH correction for multiple comparisons.

At a more fine-grained level, we tried to assess whether specific contact-wise avalanche spread differences could be identified within and across patients. In particular, we noticed that while globally the ATMs of the ON-levodopa condition show higher values with respect to the OFF-levodopa condition, there were specific edges and patients in whom we observed the opposite trend. For this reason, we used a permutation test to assess, at the patient level, which edges were significantly stronger in the ON-levodopa with respect to the OFF-levodopa conditions. For each patient, we compared the observed edge-wise difference between the ATM of the ON-levodopa and the OFF-levodopa conditions, with an edge-specific, patient-specific null distribution obtained by iteratively randomizing the labels (ON-levodopa and OFF-levodopa medication) of avalanche-specific transition matrices before averaging (Fig. 4a), see Methods. In this way, we obtained for each patient, a set of significantly stronger edges (*p* < 0.05, Benjamin-Hochberg corrected across edges) in the ON-levodopa with respect to the OFF-levodopa state, and a set of significantly stronger edges in the OFF-levodopa with respect to the ON-levodopa. We then compared edge-significance matrices across patients. First, we separated the ON > OFF significant edges from OFF > ON ones, and cumulated them over all patients (see Fig. 4b, c). As expected, a vast majority of edges were statistically significantly stronger in the ON vs. OFF states, but still a subset of patients showed stronger edges in the OFF state, in particular in cortico-cortical edges (see Fig. 4c). This analysis identifies the edges that are significantly stronger in either the ON or OFF condition for each patient. Figure 4d illustrates the maximum consensus achievable across patients for either the ON > OFF or the ON < OFF conditions, representing the highest number of patients who consistently exhibit differences in specific edges. The contrast between the blue line (ON > OFF) and the orange line (ON < OFF) indicates that the analyses are significantly more robust in the ON > OFF condition. More specifically, the y-axis represents the percentages of edges that are consistently significant across n patients (1 ≤ *n* ≤ 11), *n* is reported on the x-axis. The figure demonstrates that a specific topography of ON > OFF edges is consistently observed in nine patients out of eleven. In contrast, for the ON < OFF condition, the differences in topographies are not consistent across as many patients, with the same edges being consistent in at most two patients. We shall now proceed to the description of such topographies.

Interestingly, we found a higher number of consistently significant edges (for 8 patients) between the subthalamic nuclei contacts and motor cortex EEG channels (68.75%) than within the subthalamic nuclei (6.25%) or within the motor cortex (25.00%) in ON-levodopa with respect to OFF-levodopa condition (see Supplementary Fig. 9). This suggests that medication affects avalanches globally spreading between the STNs and the motor cortices. Furthermore, this higher prevalence of STN-cortical significant edges is consistent with our previous findings, showing that cross-modal edges seem to constitute a reliable indicator of levodopa-induced dynamic changes.

To confirm this analysis, we also computed the edge-wise group-level statistics. Specifically, we evaluated the differences in avalanche transition matrices (ATMs) for each edge across all patients between the ON-levodopa and OFF-levodopa conditions and assessed significance using the Wilcoxon signed-rank test. To correct for multiple comparisons, we applied the Benjamini–Hochberg (BH) correction. The group-level analysis was found to be consistent with the subject-level one. In particular, when considering only the edges with a significant ON > OFF difference at the group level (*p* < 0.01), 50% of them are connecting the STN contacts and the motor cortex EEG channels. A similar proportion (46.67%) of significant edges were found within the STN, while only a small fraction (3.33%) were observed within the motor cortex (see Fig. 4e). This result confirms that the differences in avalanche propagation between medication states are mainly clustered within the STN and in the interactions between the STN and the motor cortex.

Incidentally, we observed a clear asymmetry in the distribution of significant edges, with a higher proportion located in the right hemisphere. This asymmetry could be partly explained by the predominance of side-onset disease in the left hemisphere (see Supplementary Table 1). Thus, the effect of the medication would be more significant contralaterally with respect to the initially affected region, consistent with previous results on the asymmetric effects of levodopa^32^.

## Discussion

In the present work, we set out to test the hypothesis that avalanches may be affected, both in terms of statistical features and preferred spatial trajectories, by the administration of levodopa to Parkinsonian patients. This may offer a marker to track the neurophysiological changes induced by medication at the individual level in large-scale brain dynamics. Our findings confirm that neuronal avalanches occur and spread differently depending on the medication condition. In particular, while more avalanches are found in the OFF-levodopa state, they tend to spread more in the ON-levodopa state. Our results also show that differences in avalanche propagation between medication conditions involve interactions within and between the STN and motor cortex, suggesting the altered propagation of neuronal avalanches across these areas as a promising candidate for tracking the neurophysiological changes induced by levodopa at the individual level. Furthermore, we discovered that the increase in the overall propagation of avalanches is associated with motor improvement after levodopa administration. Hence, the L-Dopa-induced changes in the ATMs might be related to the therapeutic response.

Our findings indicate a higher frequency of avalanches in the OFF-levodopa state compared to the ON-levodopa state and, conversely, a higher inter-avalanche interval durations in the ON-state. On the one hand, this finding is reminiscent of the known pathological hypersynchronization that is present in Parkinsonian patients and more evident without therapy^33–35^. On the other hand, we found a reduced number of avalanches, lasting longer and being larger in size in the ON-levodopa state. This analysis may hint to an opposing pattern between neuronal avalanches and beta bursts in response to levodopa medication. While levodopa reduces the number of avalanches and increases their duration, it is known from the literature that it also reduces β burst probability, increases the number of short β bursts, and decreases the number of long β bursts^16^. Understanding the interaction between these two metrics could provide valuable insights into how PD affects brain dynamics and may serve as a foundation for future research. Therefore, our finding suggests that the picture might be more complex than previously thought, with changes in the dynamical structure hardly reported using a conventional oscillatory perspective.

Despite the lower number of neuronal avalanches in the ON-levodopa state, their spreading, once they occur, appears to be facilitated by the presence of levodopa, as evident from the generally higher transition probabilities in the ON state. This result demonstrates that neuronal avalanches are more likely to spontaneously propagate in the ON-levodopa condition for Parkinsonian patients. Conversely, while more avalanches start during the OFF state, they fail to effectively recruit a high number of brain areas. Furthermore, we show that on closer topographical analysis, the differences between the conditions are not equally present throughout the brain but, rather, they mainly involve the interaction between the STN and the motor cortex in the sense of more spreading of avalanches taking place under levodopa.

To assess that our findings are not entirely driven by the power in the lower-frequencies (i.e., theta and alpha), we reanalyzed the data after filtering it in the alpha and theta bands. The averages of the ATMs were no longer significantly different between ON and OFF states (Supplementary Fig. 10), although a trend can be seen for the cortical edges in the alpha band. Furthermore, they did not correlate with clinical improvement (Supplementary Figs. 11, 12). These results make it unlikely that our findings are driven by the power of the lower frequency bands alone.

Our results align with previous neuroimaging research indicating that in Parkinson’s disease, dopaminergic depletion is linked with malfunctioning of the basal ganglia motor circuit (BGMC)^36–39^, along with reduced connectivity of the striato-thalamo-cortical motor pathways^40–42^. In addition, prior neuroimaging research has indicated that the administration of levodopa might partially restore the abnormal functional connectivity (FC) in the BGMC circuit among patients with Parkinson’s disease. For instance, it may achieve this by boosting neural activity within the supplementary motor area and striatum^36,43–47^ and reestablishing connectivity in the striato-cortical motor pathway^48,49^. The restoration of striatal-cortical connectivity may result in restoration of cortico-subthalamic connectivity as observed in our results via the indirect pathway.

Furthermore, we demonstrated that the increase in the overall propagation of avalanches correlates with motor improvement after levodopa administration. This suggests that the proposed pipeline, beyond tracking neurophysiological changes related to the administration of levodopa, might provide a readout to the clinical improvement of the patients. This is aligned with previous studies, which showed a correlation between changes in FC and improvements in clinical symptoms following levodopa treatment^48–52^. In addition, the correlation between the ratio of the average ATMs in the ON and the OFF states with the clinical improvement after levodopa administration reached significance when computed selectively on the STN-cortex edges. This might indicate that clinical improvement relates the most to the coordination of activities between the STN and the motor cortex. These results complement the evidence provided above about the differences in avalanche propagation between medication states. Despite some variability in clinical presentations, the trends in individual patient differences (see Fig. 2c–f) and clinical correlations (see Fig. 3a) remained remarkably consistent across patients. However, larger samples may be needed to investigate the effect of different clinical forms.

Of note, the reported differences of the propagation of avalanches are observed not only at the subcortical level through STN activity but also at the cortical level through EEG activity, which could be relevant, for example, for clinical purposes, given the ease of access to EEG data. We also found a significant increase in the propagation of avalanches for both short-range (within the subthalamic nuclei and within the motor cortex) and long-range (between the subthalamic nuclei and the motor cortex) connections in the ON-levodopa state compared to OFF-levodopa state. Also, this means that the effects can be observed within a modality as well as between modalities. To our knowledge, there are no previous works that have studied the probability of propagation of avalanches across modalities.

In summary, our study revealed that the administration of Levodopa enhanced the ease of propagation of neuronal avalanches between the motor cortex and the STN. This aligns with prior studies suggesting that treatment using levodopa partially restores the function of the basal ganglia motor circuit^36,43–47,51,52^. Additionally, we found that the increase in the overall propagation of avalanches is correlated with motor improvement after levodopa administration. Therefore, proposing the ATM as a potential biomarker serves not only to track the medication’s effect but also to track the individual clinical improvement in Parkinson’s disease. Furthermore, and more generally, our study adds to the growing literature showing that conceptualizing brain dynamics in PD in terms of aperiodic bursts yields relevant information that might not be apparent in a more oscillatory perspective. Therefore, our findings provide insights into the neural mechanisms underlying the effect of levodopa therapy on the interaction between the STN and the motor cortex in Parkinson’s disease.

While our study provides valuable insights and contributes to the field of Parkinson’s disease research, several limitations should be considered.

First, although our statistical analyses rely on fixed sensor positions, we did not co-register the sensor positions to each patient’s MRI. However, EEG placement followed standard anatomical landmarks based on the 10–20 international EEG system. Nonetheless, we acknowledge that electrode positioning may differ slightly across patients. To address this, we conducted the analyses at the individual level, contrasting the data within each individual. Additionally, we compared the results across individuals to identify consistent patterns of sensorimotor area activities.

Second, we analyzed the DBS contacts without distinguishing between those in motor and non-motor regions of the STN. To assess the robustness of our results to the specific setup, we averaged all deep contacts for each hemisphere and recomputed the ATMs. The differences between ON and OFF remained consistent in this additional analysis (see Supplementary Fig. 7). However, this study lacks more precise information about the location of each contact with respect to the subregions of the STN that are part of a network that includes the motor cortices. While the average analysis showed robustness of our results, a more precise spatial resolution is warranted to further validate our results.

Third, our findings should be interpreted with caution due to the relatively small sample size (11 PD patients).

Fourth, the long-lasting effects of dopamine agonists represent another potential confounder. These medications exert effects beyond that of levodopa, with residual influences persisting into the OFF-medication condition. This could potentially confound observed differences between ON- and OFF-states. Future studies could consider strategies to better isolate the specific effects of levodopa.

Finally, signal leakage may impact our results, particularly affecting nearby regions more than distant ones (e.g., STN to cortex). While the time shift used in ATM calculations may help mitigate this issue, it cannot entirely eliminate its influence. Future work could explore advanced methods to further minimize the effects of signal leakage. Furthermore, the consistency observed at the subject-level is reassuring in this regard (as the geometry of the head is constant in this experimental design).

## Methods

### Participants, surgery, experiments, and data collection

The study involved 11 patients diagnosed with advanced Parkinson’s disease who underwent deep brain stimulation (DBS) surgery targeting the subthalamic nucleus (STN). Recordings were taken using Deep Brain Stimulation leads and EEG electrodes placed bilaterally over the motor areas. The recordings were performed under two conditions: OFF-levodopa medication (before its administration) and ON-levodopa medication (after its administration). The order of the conditions has been balanced in the cohort. The ON-medication recordings took place 45 min after levodopa intake, with the ON-state confirmed through a clinical examination using the MDS-Unified Parkinson’s Disease Rating Scale (MDS-UPDRS). The clinical information of the patients is reported in the Supplementary information (Supplementary Table 1). The local ethics committee (Comité de Protection des Personnes (CPP) Sud Méditerranée I) approved the study (registration number RCB: 2009-A00913-54), and all participants provided written informed consent. Bilateral DBS surgery was performed using Medtronic 3389 DBS leads. Intra-operative micro-recordings and macro-stimulation during surgery were utilized for accurate lead placement, and postoperative clinical assessments, supported by the fusion of preoperative MRI and postoperative CT scans for validation, were performed. The experimental procedure involved temporary externalization of DBS electrodes before connecting them to the implantable pulse generator, usually five days later. Patients abstained from taking dopaminergic medication the night before recordings. Brain signals were recorded for 2–3 min with patients at rest, seated comfortably. Simultaneous recordings of local field potentials (LFPs) and scalp EEG signals were obtained, including 8 LFP contacts for the subthalamic nuclei (4 for the Left STN and 4 for the Right STN), and 6 EEG channels in the bilateral motor areas (F3, Fz, F4, C3, C4, Cz). A common average reference was used for both the LFP and EEG signals during recording. Spike artifacts were minimized and removed using the Spike2 Software. Data was imported into Matlab, with signal durations ranging from 119.2 s to 155.5 s and a mean duration of 127.2 s ± 2.5. Our study uses a dataset that partially overlaps with the one used in ref. ^30^.

### Data preprocessing

Signal preprocessing was performed using the Fieldtrip toolbox^53^. The continuous signal was first high-pass filtered at 1.3 Hz and low-pass filtered at 45 Hz using a Hamming window, a two-pass direction, and a Butterworth filter. Subsequently, the data was down-sampled to 512 Hz. The signal underwent visual inspection to remove noisy segments.

Since different duration of the data might affect the results, we carried out a paired t-test, which did not show any statistical difference (d = 0.13, *p* = 0.663; d represents the effect size, calculated using Cohen’s d; see Supplementary Fig. 13). We also repeated the analysis using equal data lengths within each patient, and the findings remained consistent (see Supplementary Fig. 14). The only exception was the avalanche rate, which did not show a significant *p*-value (paired t-test:d = 0.64, *p* = 0.058; see Supplementary Fig. 14a). However, the overall pattern remained consistent.

### Estimation of neuronal avalanches

Neuronal avalanches were estimated by binarizing z-scored activities (each brain signal, including EEG and LFP, was independently z-scored across time) to identify salient events in neural dynamics that exceeded a threshold of 2 standard deviations (|z| = 2). When a brain signal has a z-score above this threshold, it is classified as active (assigned a value of 1), while all other time points are considered inactive (assigned a value of 0). A neuronal avalanche initiates when, in a sequence of consecutive time bins, at least one channel becomes active (|z| > 2) and concludes when all channels return to an inactive state^19,54^. Our results remained robust across threshold variations within the range of 1.7–2.7 standard deviations (see Supplementary Fig. 15).

Neuronal avalanches are characterized by their size, *s*, defined as the number of channels recruited during the avalanche. They can also be characterized by the duration, and the inter-avalanche interval (IAI), defined as the time interval between two consecutive avalanches.

Our prominent interest was to assess the effect of levodopa medication on neuronal avalanches features, such as their number, size, duration, as well as the inter-avalanche interval. To achieve this, we compared the avalanche rate (i.e., the number of avalanches per second). At the subject level, we used the Mann–Whitney test to assess the difference in the distributions of the number of avalanches across 4-second segments between ON and OFF conditions. At the group level, we employed the paired t-test to assess the significance of the avalanche rate per second in ON-levodopa versus OFF-levodopa conditions.

We also compared the size, duration, and inter-avalanche interval between ON-levodopa and OFF-levodopa conditions at both the subject and group levels. At the subject level, we used the two-sample Kolmogorov–Smirnov (K-S) test to assess the significance of the differences in the distributions of size, duration, and inter-avalanche interval between ON-levodopa and OFF-levodopa conditions. At the group level, we computed the average values for each avalanche feature (size, duration, and inter-avalanche interval) per subject for each condition. Then, we conducted a paired t-test to compare the distributions of these features between the ON and OFF conditions. The reason behind choosing the K-S test for subject level analysis is its lack of gaussianity assumptions, given the non-linear, fat-tailed nature of avalanche dynamics^54^.

### Avalanche transition matrix

We calculated an avalanche-specific transition matrix (TM) where rows and columns represent channels. The element (i, j) of this matrix represents the probability that channel j was active at time t + 1, given that channel i was active at time t (the ij-th entry). Thus, the edge between channels i and j indicates the probability of these two channels being sequentially recruited by an avalanche. We excluded avalanches shorter than 2 samples in the calculation of the avalanche transition matrix, as these were considered likely to represent noise rather than meaningful neuronal activity. For each patient, we obtained an average transition matrix by averaging edge-wise over all avalanches and then symmetrized it^31^. Therefore, we obtained two symmetric ATMs per patient, one corresponding to the ON condition and the other to the OFF condition.

### Statistical analysis

For each patient, we compared the ATMs in two conditions: ON-levodopa medication and OFF-levodopa medication. This comparison provides insight into the probabilities of perturbations spreading across two brain regions. To confirm this statistically, we randomly shuffled the labels of the avalanche-specific transition matrices for each individual. We repeated this procedure 10,000 times, obtaining, for each edge, the distribution of differences under the null hypothesis that the transition matrices would not capture any difference between the two medication conditions. Note that this method does not require normality of the initial distributions. These distributions were then used to identify edges that showed significant differences between the two medication states at the subject level. The *p*-values were calculated as the proportion of random differences greater than the observed difference. The significance levels were corrected for multiple comparisons across edges using the Benjamini–Hochberg (BH) correction. Following this procedure, we obtained a matrix for each patient containing the edges that exhibited significant differences between the two conditions. After this step, our focus shifted to edges that were consistently significant across patients for both the ON-levodopa medication and OFF-levodopa medication conditions. This same method was previously employed by Corsi et al.^55^.

We also explored group-level analysis in addition to within-subject analyses. Specifically, we evaluated the differences in avalanche transition matrices (ATMs) for each edge across all patients between the ON-levodopa and OFF-levodopa conditions and assessed significance using the Wilcoxon signed-rank test. To correct for multiple comparisons, we applied the BH correction.

We also conducted additional analyses at the group level to assess whether our results were driven specifically by edges computed between electrodes belonging to the same/different modality (EEG or deep electrodes), we classified the edges into three groups: *STN-STN*, edges connecting two channels both located in the STN (recorded using deep stimulation electrodes), *cortex-cortex*, edges connecting two channels located both in the motor cortex (recorded using EEG), and *STN-cortex*, that is edges connecting one channel located in the STN with one located in the cortex. Next, we compared the average of each group of edges between the ON-levodopa and the OFF-levodopa conditions at the population level. To assess the significance of the differences, we conducted a Wilcoxon signed-rank test. This procedure was chosen to confirm the significance of the differences in the propagation of avalanches between ON-levodopa and OFF-levodopa states in both short-range connections (within the STN, within the motor cortex) and long-range connections (between the STN and the motor cortex) at the group-level, and to assess whether these differences are modality-independent.

### Clinical correlation analysis

To establish a relationship between the change in the overall propagation of avalanches and clinical improvement after levodopa administration, we assessed the relationship between the clinical improvement and the ratio of the average of ATMs ON and OFF levodopa conditions. This analysis was conducted on ten of the eleven patients, excluding patient number eight due to unavailable clinical data. The clinical improvement was defined as follows^16^:

$${\bf{Clinical}}\_{\bf{improvement}}=(({\bf{updrsIII}}\_{\bf{off}}-{\bf{updrsIII}}\_{\bf{on}})/{\bf{updrsIII}}\_{\bf{off}})* {\bf{100}}$$

Where ‘updrsIII_off’ and ‘updrsIII_on' refer to the Unified Parkinson’s Disease Rating Scale Part III before and after medication intake, respectively.

We utilized robust linear regression to obtain more accurate estimates of regression coefficients, reducing the influence of the outliers or deviations from the linear relationship between variables. Specifically, we utilized the “robustfit.m” MATLAB function^56^, with the bisquare fitting weight function. This function provides coefficient estimates and model statistics as outputs.

We utilized the same technique (i.e. Robust linear regression) to assess the relationship between the ratio of the average ATMs in the ON and the OFF states, computed over three different types of edges (cortex-cortex, STN-cortex, and STN-STN), with the clinical improvement after levodopa administration. In this case, before computing the ratios, we averaged the ATMs ON and the ATMs OFF only considering the edges connecting two channels both located in the cortex for cortex-cortex edges, or edges hinging on the STN and the cortex for STN-cortex edges, and edges connecting two channels both located in the STN for STN-STN edges.

## Supplementary information

Supplementary_information_L-Dopa_alters_aperiodic_bursts_in_PD

## Data Availability

Due to the clinical nature of the data, it can’t be publicly available.

## Code availability

The codes employed in this study are available at https://github.com/Hasnae12/neuronal_avalanches_PD.
