# Supplementary material for: L-Dopa-induced changes in aperiodic bursts dynamics relate to individual clinical improvement in Parkinson’s disease
Source: NPJ Parkinsons Dis. 2025 Jun 10;11:158. doi: 10.1038/s41531-025-01024-w (PMC12152154; doi:10.1038/s41531-025-01024-w)
Supplement: Supplementary file 1 — Supplementary_information_L-Dopa_alters_aperiodic_bursts_in_PD [file 41531_2025_1024_MOESM1_ESM.pdf]

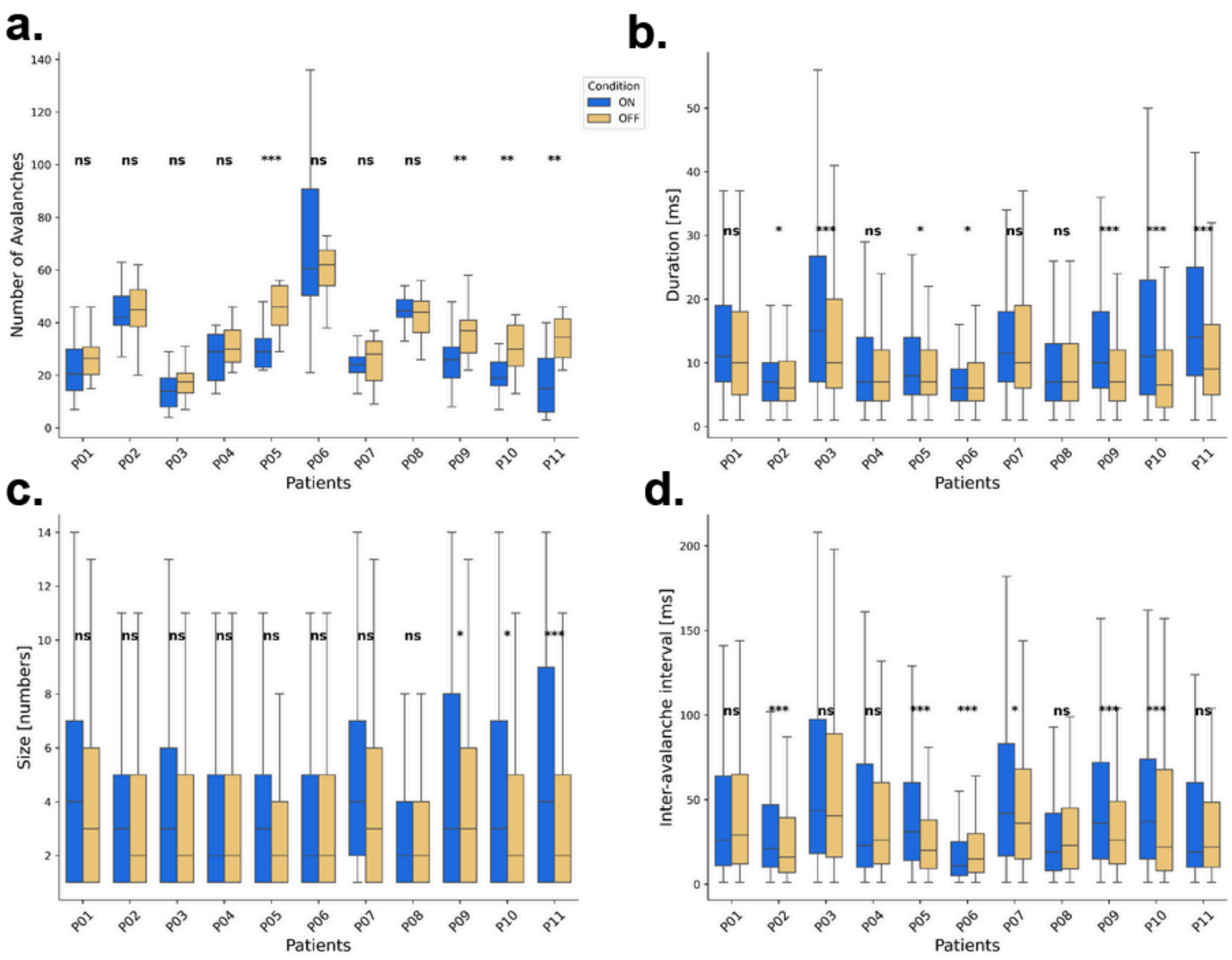

***Supplementary Figure 1. Subject-level analysis of avalanches features. (a) Total number of avalanches per segment of 4 seconds**, individual comparisons in the ON-levodopa versus OFF-levodopa states for each patient. The significance of the difference in the distributions of the number of avalanches across 4-second segments ON versus OFF was assessed using the Mann-Whitney test. **(b) Avalanche durations** (in ms), individual comparisons. **(c) Avalanche size (# of channels)**, individual comparisons. **(d) Inter-avalanche intervals** (ms), individual comparisons. For b-c-d, we used the two-sample Kolmogorov-Smirnov (K-S) test to assess the significance of the differences in the distributions of size, duration, and inter-avalanche interval between ON-levodopa and OFF-levodopa conditions. The significance level are indicated as *** : p< 0.001, ** : p<0.01, *: p<0.05, ns = non-significant, p refers to p-value.*

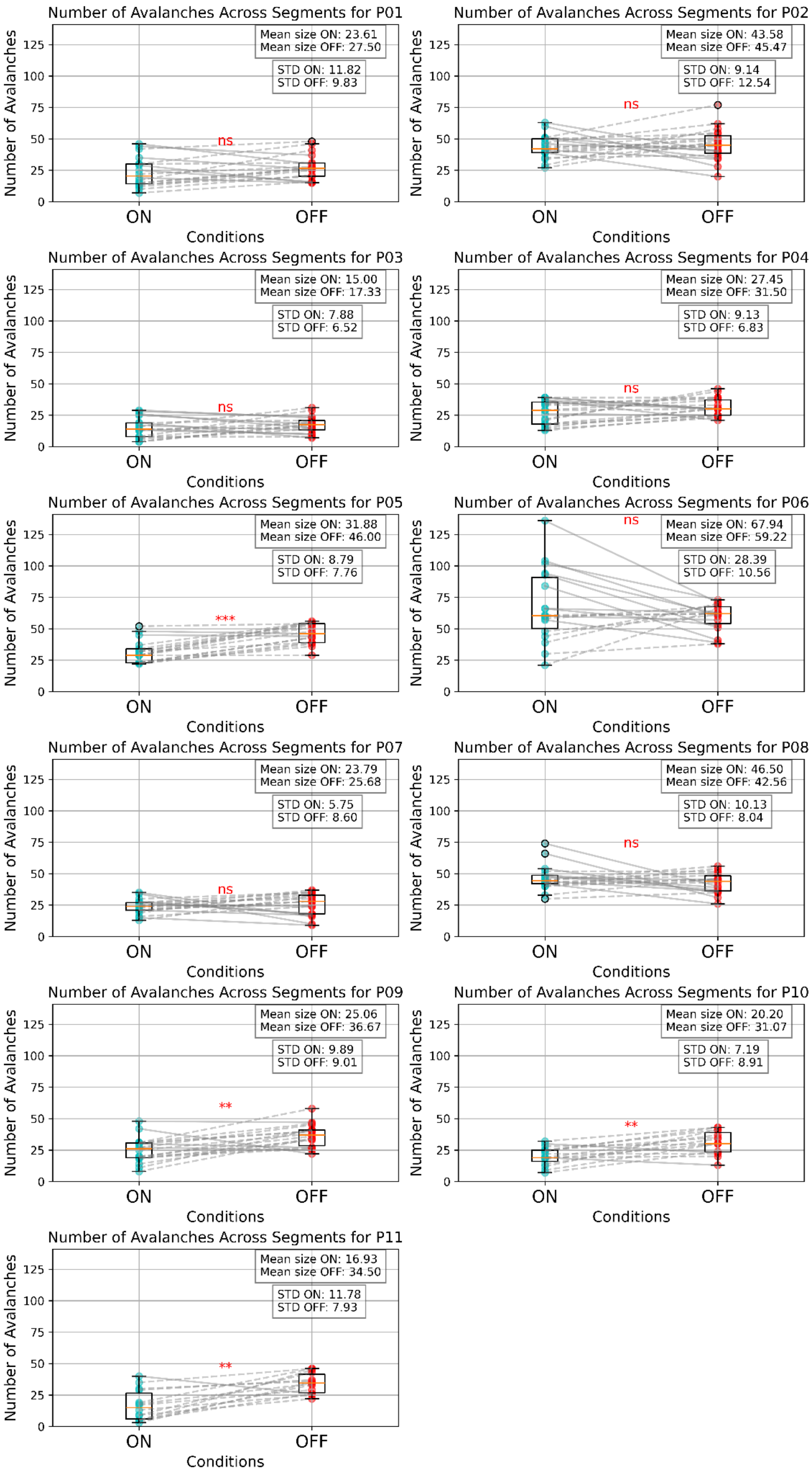

Supplementary Figure 2. Subject level analysis of avalanches features. *Comparison of the number of avalanches across 4-second segments for each patient in the ON-state and OFF-state, along with Mann-Whitney test results, mean values, and standard deviations. Dashed lines indicate higher values in the OFF condition compared to ON, while solid lines represent higher values in the ON condition compared to OFF. The significance level are indicated as *** : p< 0.001, ** : p<0.01, *: p<0.05, ns = non-significant, p refers to p-value.*

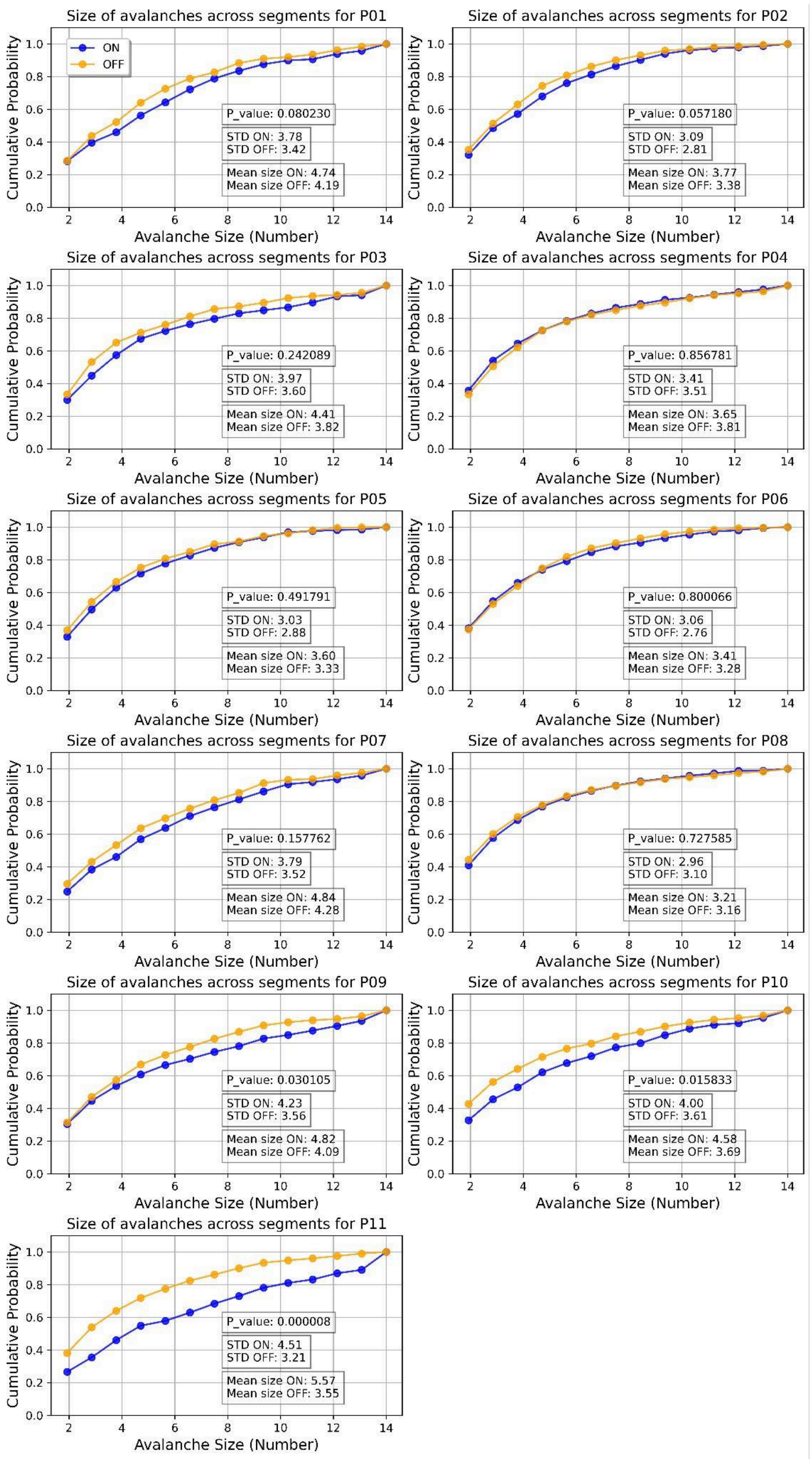

Supplementary Figure 3. Subject level analysis of avalanches features. *Comparison of the cumulative distribution functions of avalanche sizes for each patient in the ON-state and OFF-state, along with Kolmogorov-Smirnov (K-S) test results, mean values, and standard deviations.*

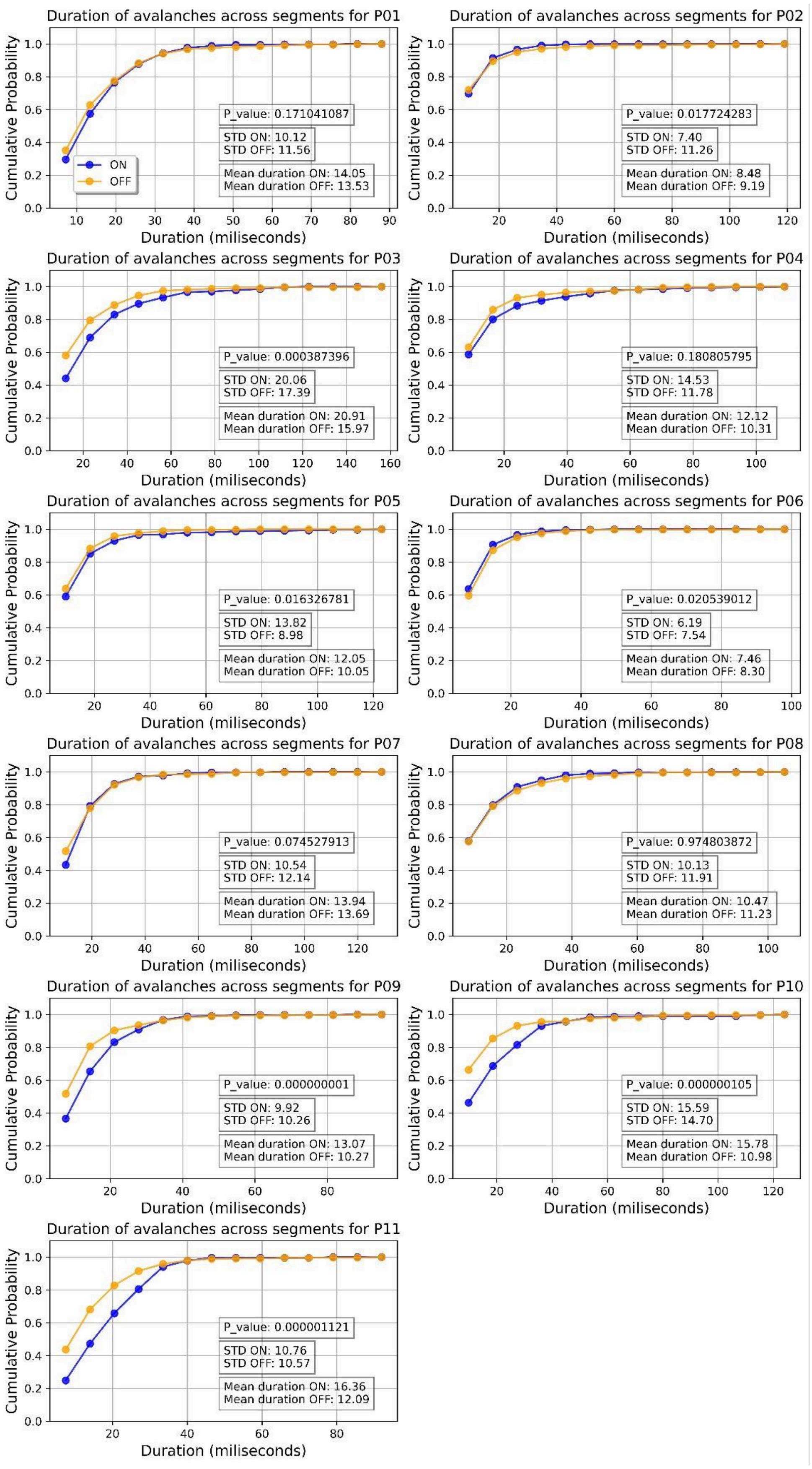

Supplementary Figure 4. Subject level analysis of avalanches features. *Comparison of the cumulative distribution functions of avalanche durations for each patient in the ON-state and OFF-state, along with Kolmogorov-Smirnov (K-S) test results, mean values, and standard deviations.*

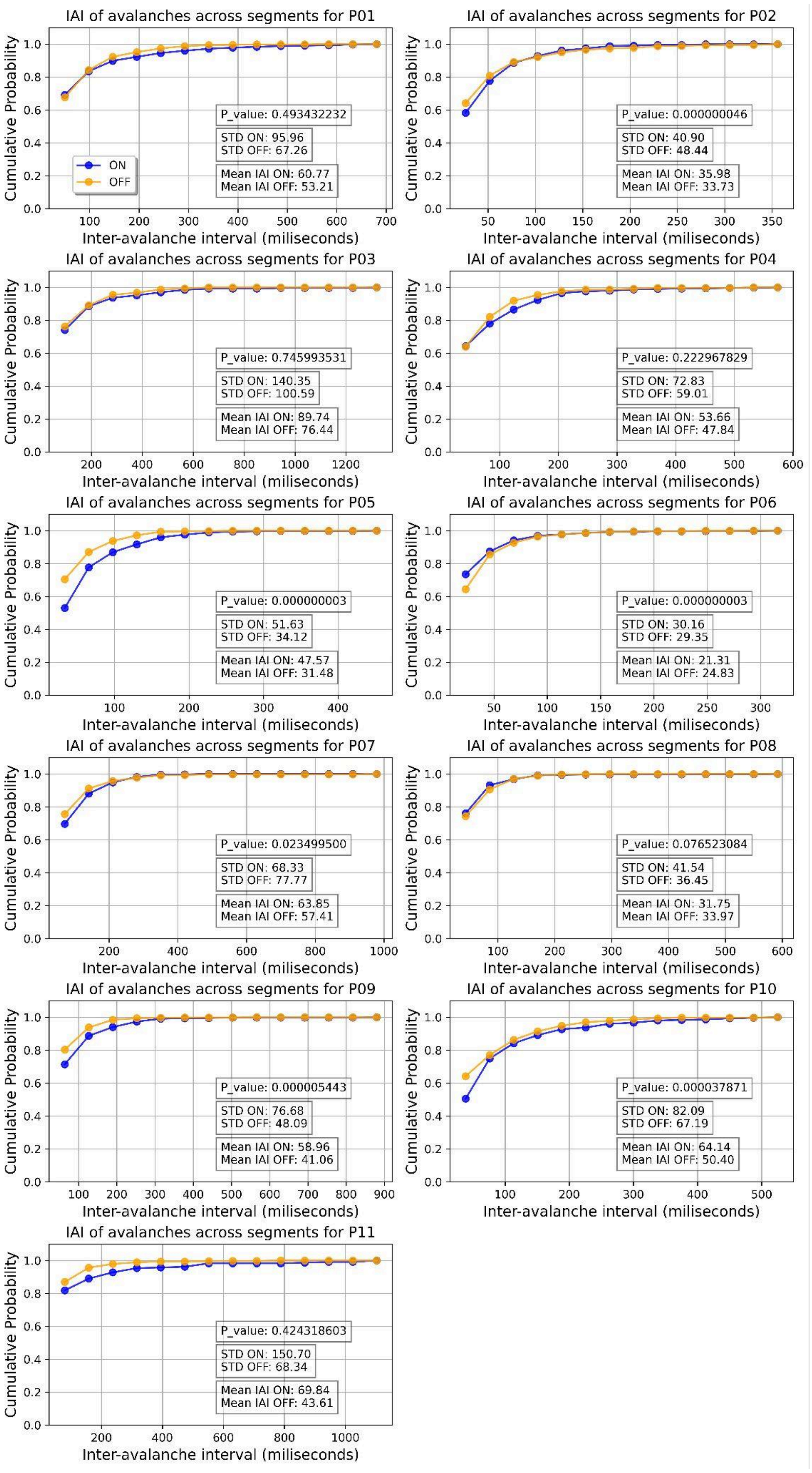

Supplementary Figure 5. Subject level analysis of avalanches features. *Comparison of the cumulative distribution functions of Inter-avalanche intervals (IAI) for each patient in the ON-state and OFF-state, along with Kolmogorov-Smirnov (K-S) test results, mean values, and standard deviations.*

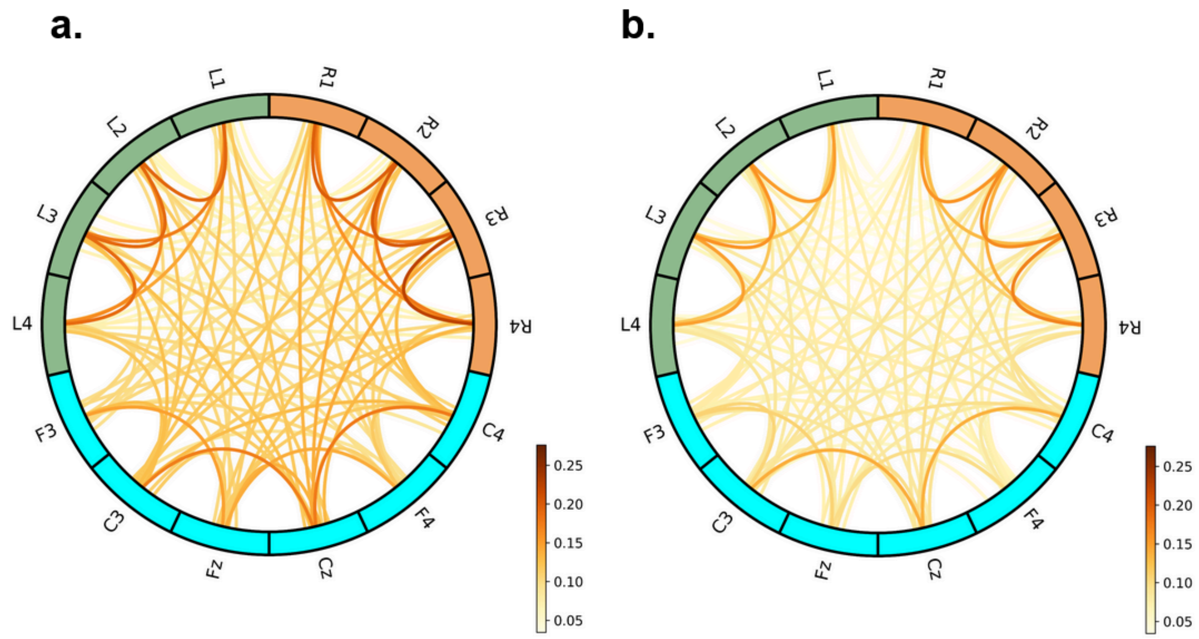

Supplementary Figure 6. Average of the original Avalanche Transition Matrices (ATMs) across patients in ON-levodopa and OFF-levodopa conditions. *In* ***(a),*** *we plotted the average ATMs in the ON-levodopa across all patients. In* ***(b),*** *we plotted the average ATMs in the OFF-levodopa across all patients. We represented the brain as a network, where channels are nodes linked by edges. For the nodes, the green represents the left LFP contacts, orange represents the right LFP contacts, and blue represents the EEG channels.*

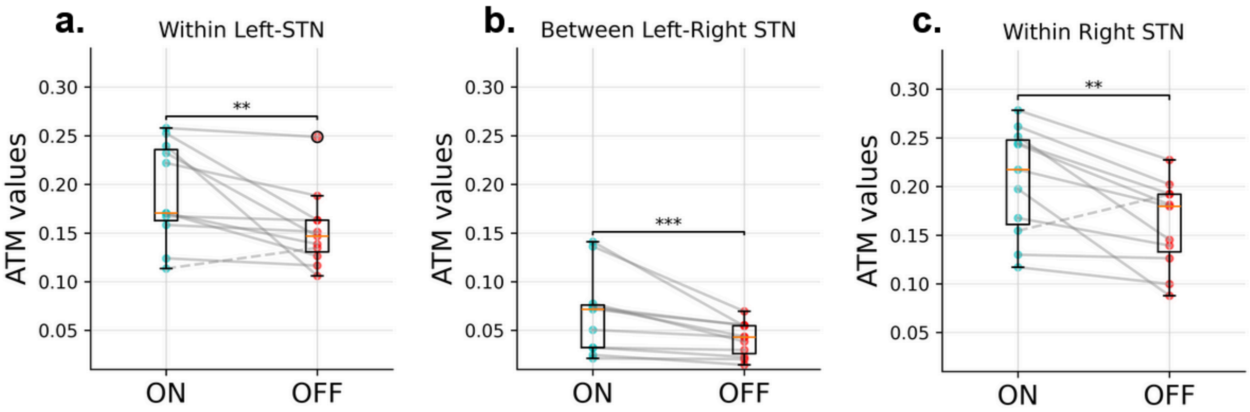

Supplementary Figure 7. ATM Analysis: Within vs. Between STNs. (a) Within Left STN*: ATM differences between ON and OFF conditions within the left STN.* ***(b) Between Left and Right STNs****: ATM differences between STNs of different hemispheres.* ***(c) Within Right STN****: ATM differences between ON and OFF conditions within the right STN. Dashed lines indicate higher values in the OFF condition compared to ON, while solid lines represent higher values in the ON condition compared to OFF. A Wilcoxon signed-rank test was used, resulting in the following Wilcoxon effect sizes (denoted by r) and p-values (denoted by p) : within the left STN (r=0.75, p=0.0097), between the left and right STNs (r=0.88, p=0.00097), and within the right STN (r=0.77, p=0.0068).*

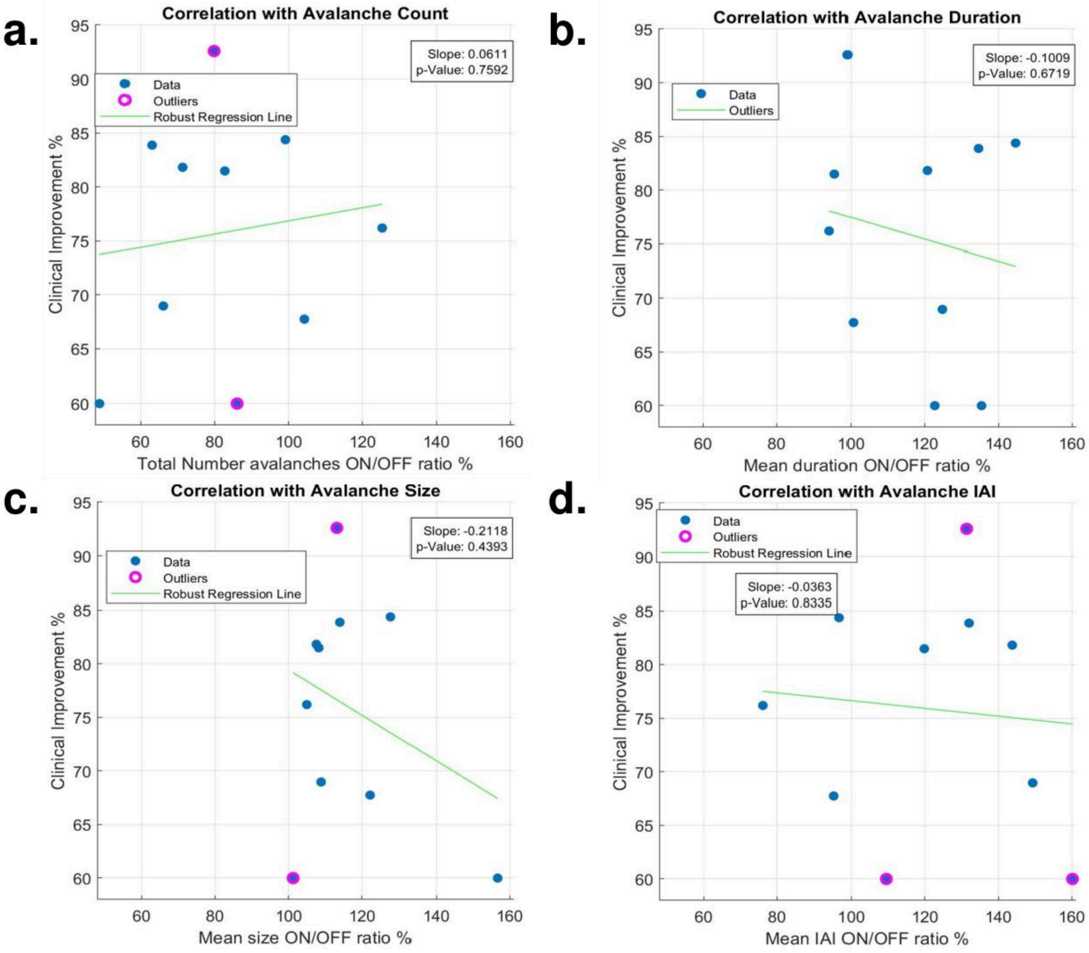

Supplementary Figure 8. Correlation analysis with avalanche features. *Robust linear regression. Each patient is represented as a blue dot, except for the outliers with a pink circle. The regression coefficients (denoted by r), p-values (denoted by p).* ***(a) Clinical correlation with the total number of avalanches*** *(r=0.06, p=0.75).* ***(b) Clinical correlation with the average duration*** *(r=-0.10, p=0.67).* ***(c) Clinical correlation with the average size*** *(r=-0.21, p=0.43).* ***(d) Clinical correlation with the average inter-avalanche interval (IAI)*** *(r=-0.03, p=0.83).*

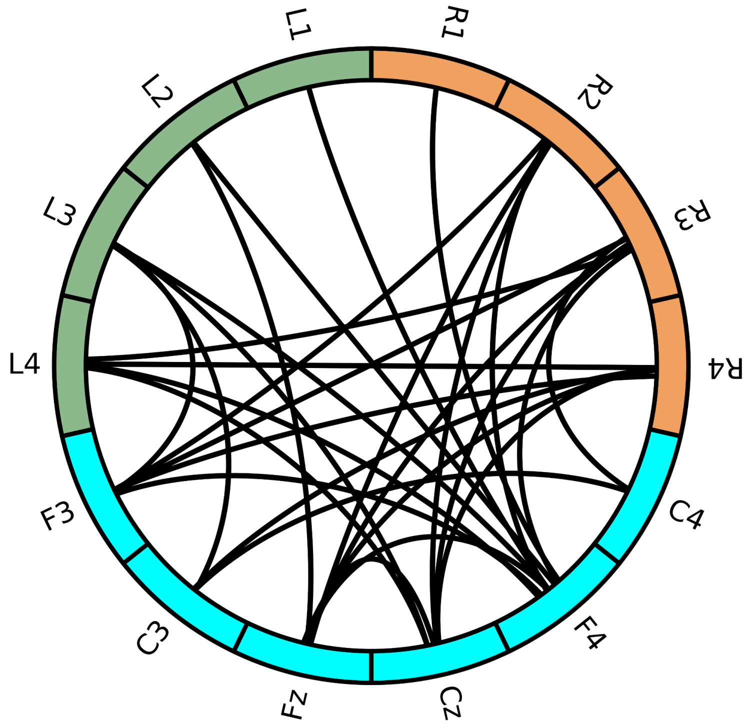

***Supplementary Figure 9. Edges with cumulative consistency across 8** patients in the ON>OFF case.*

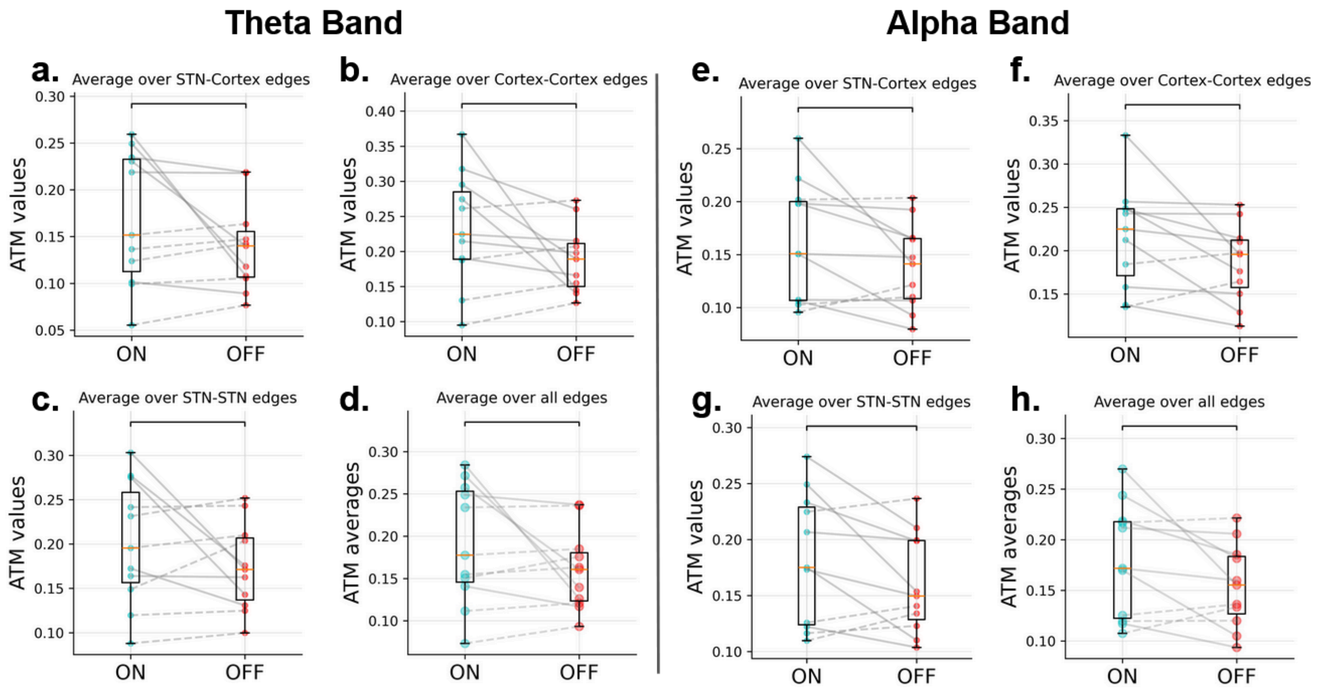

Supplementary Figure 10. ATM in ON- and OFF-levodopa conditions after filtering the data in theta and alpha bands. (a) Group-level STN-Cortical edge average comparison *(r=0.24, p=0.46),* ***(b) Group-level Cortico-Cortical edge average comparison*** *(r=0.37, p=0.24),* ***(c) Group-level STN-STN edge average comparison*** *(r=0.13, p=0.70),* ***(d) Group-level ATM average comparison for the theta band*** *(r=0.24, p=0.46).* ***(e), (f), (g),*** *and* ***(h)*** *correspond to* ***(a), (b), (c), and (d)****, respectively, but with data filtered in the alpha band. Wilcoxon effect sizes (denoted by r) and p-values (denoted by p) are:* ***(e)*** *r=0.53, p=0.08;* ***(f)*** *r=0.58, p=0.053;* ***(g)*** *r=0.48, p=0.12;* ***(h)*** *r=0.50, p=0.10. Dashed lines indicate higher values in the OFF condition compared to ON, while solid lines represent higher values in the ON condition compared to OFF.*

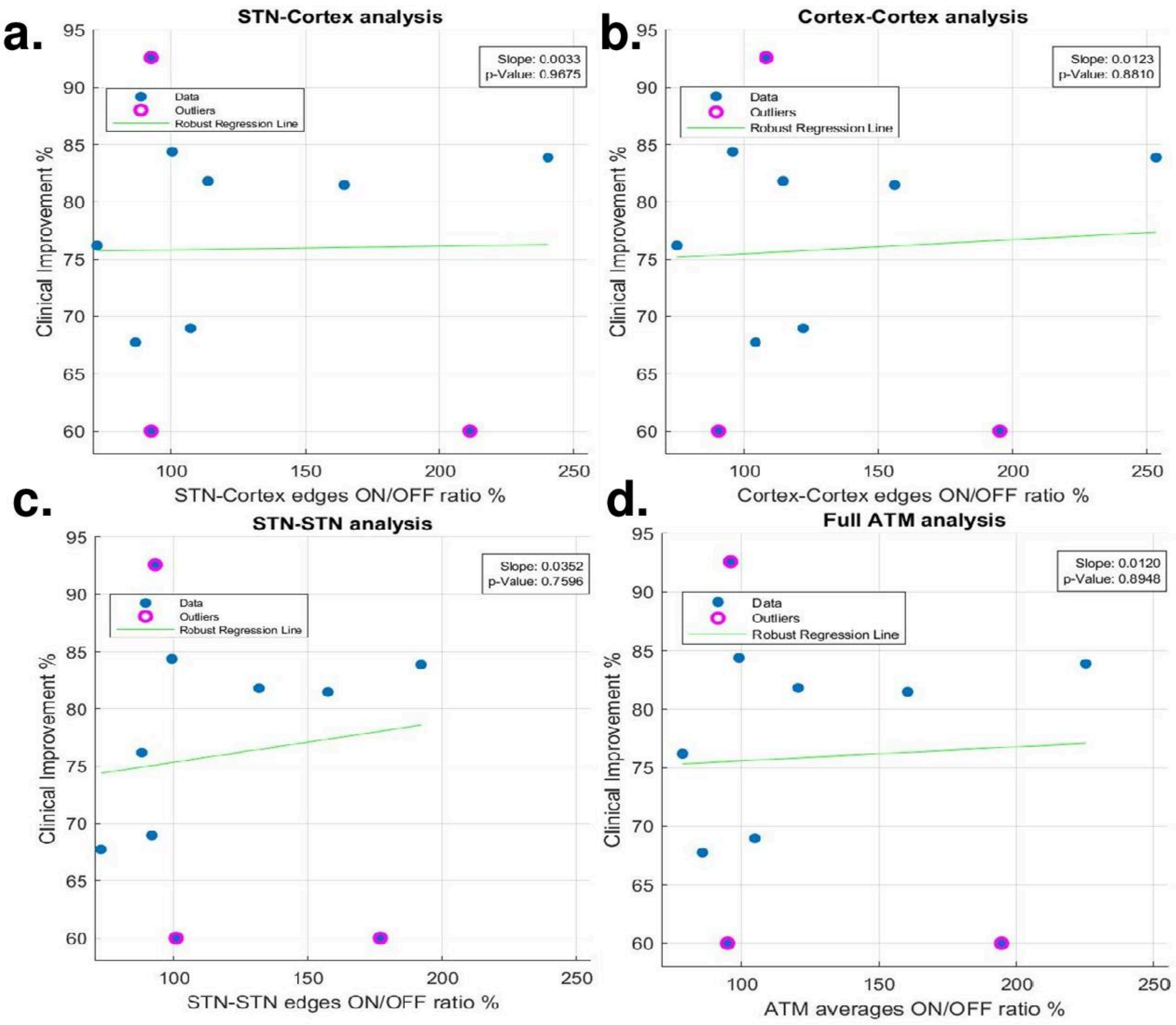

Supplementary Figure 11. Correlation analysis after filtering the data in theta band. *Robust linear regression. Each patient is represented as a blue dot, except for the outliers with a pink circle. The regression coefficients (denoted by r), p-values (denoted by p).* ***(a) STN-Cortex edges ON/OFF ratio vs. clinical improvement*** *(r=0.003, p=0.96).* ***(b) Cortex-Cortex edges ON/OFF ratio vs. clinical improvement*** *(r=0.01, p=0.88)****. (c) STN-STN edges ON/OFF ratio vs. clinical improvement*** *(r=0.03 , p=0.75).* ***(d) ATM averages ON/OFF ratio vs. clinical improvement*** *(r=0.01, p=0.89)* ***for the theta band****.*

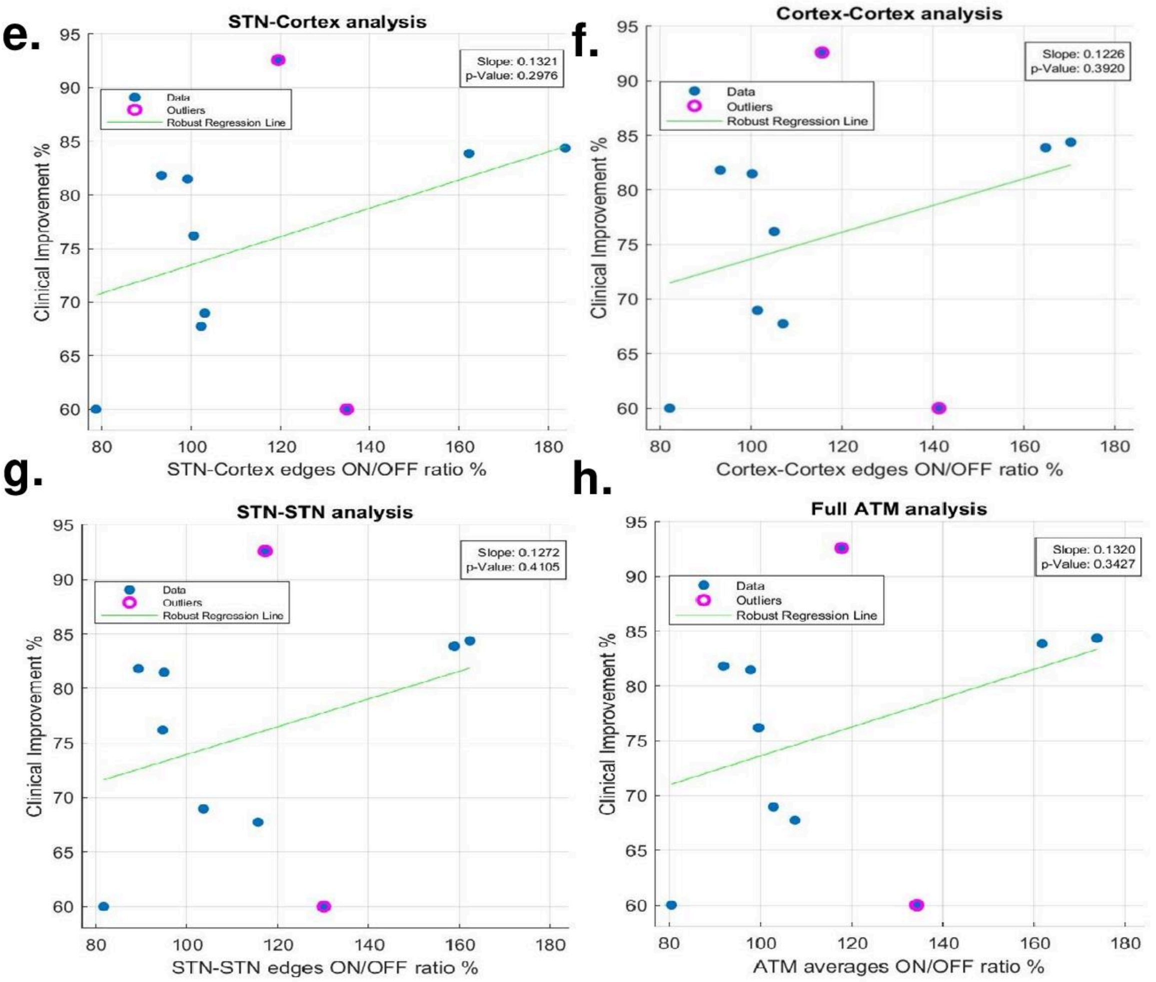

Supplementary Figure 12. Correlation analysis after filtering the data in alpha band. *Robust linear regression. Each patient is represented as a blue dot, except for the outliers with a pink circle. The regression coefficients (denoted by r), p-values (denoted by p).* ***(e) STN-Cortex edges ON/OFF ratio vs. clinical improvement*** *(r=0.13, p=0.29).* ***(f) Cortex-Cortex edges ON/OFF ratio vs. clinical improvement*** *(r=0.12, p=0.39)****. (g) STN-STN edges ON/OFF ratio vs. clinical improvement*** *(r=0.12, p=0.41).* ***(h) ATM averages ON/OFF ratio vs. clinical improvement*** *(r=0.13, p=0.34)* ***for the alpha band.***

Patient	Age Range (years)	Sex	Disease Duration (years)	First symptom	Side onset	UPDRS III OFF	UPDRSIII ON	Delay between surgery and recordings (days)	Medication
P01	51-55	M	14	Tremor	R	27	5	3	ropinirole 8mg; l-dopa 1250mg/j ; entacapone ; rasagiline
P02	66-70	F	12	AR	R	31	10	3	ropinirole 8mg; l-dopa 700mg/j ; entacapone
P03	66-70	M	10	Tremor	R	32	5	3	piribedil 150mg/j ; l-dopa 1250mg/j ; entacapone
P04	51-55	F	8	Tremor	L	40	16	3	
P05	51-55	F	10	AR	L	11	2	3	ropinirole 16mg ; l-dopa 400mg
P06	61-65	F	12	AR	L	21	5	3	ropinirole 20mg; ldopa 725mg; entacapone; rasagiline 1mg
P07	56-60	M	17	AR	R	27	2	3	pramipexole 2,10mg; ldopa 1200mg; entacapone
P09	61-65	M	14	Tremor	L	29	9	3	piribedil 150mg; ldopa 900mg, entacapone
P10	61-65	M	7	Tremor	L	31	5	3	ropinirole 24mg; ldopa 800mg; entacapone; rasagiline 1mg
P11	56-60	F	8	Tremor	L	20	8	5	priribedil 150mg; ldopa 1000mg; entacapone

Supplementary Table 1. Clinical features of some patients. *F = female; M = male; L = left; R = right; AR=Akinetic rigid; UPDRS= Unified Parkinson's Disease Rating Scale. Clinical data from 10 PD patients shows a reduction in the UPDRS after the intake of medication, indicating a positive response to medication.*

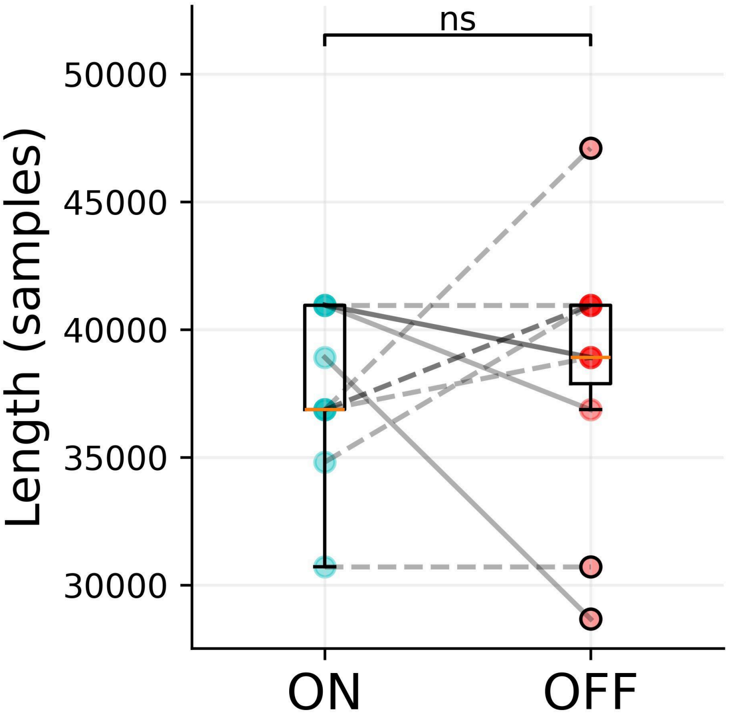

Supplementary Figure 13. Difference in Length of Preprocessed Data. *We assessed the significance of the difference in preprocessed data lengths between the ON and OFF medication states at the group level using a paired t-test. This analysis resulted in an effect size, measured as Cohen's d, of 0.13 and a p-value of 0.663. Dashed lines indicate higher values in the OFF condition compared to ON, while solid lines represent higher values in the ON condition compared to OFF. 'ns' denotes a non-significant p-value.*

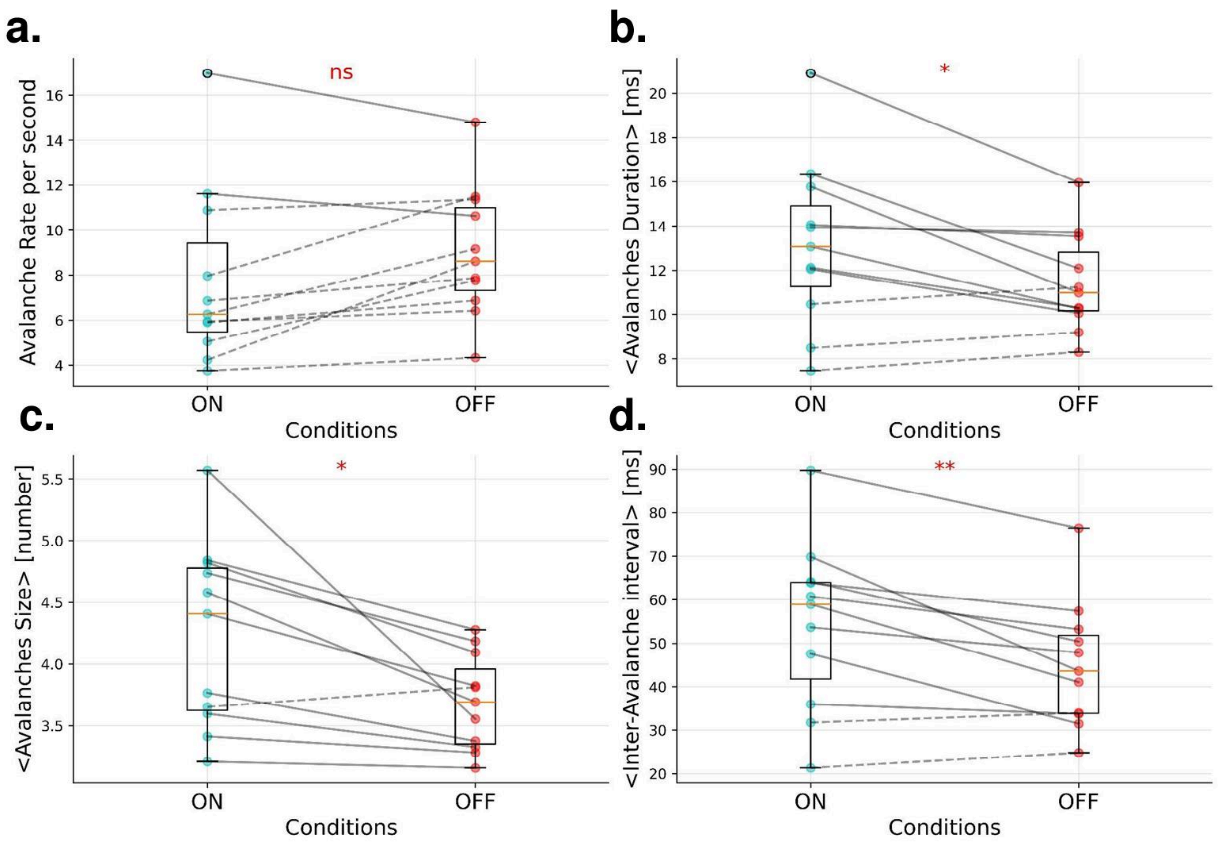

Supplementary Figure 14. Group-level analysis of avalanche features using equal data lengths within each patient. (a) Avalanche Rate Comparison: *Comparison of avalanche rate per second between ON- and OFF-levodopa conditions at the group level.* ***(b) Avalanche Duration:*** *Group-level comparison of average avalanche duration (ms) for each patient.* ***(c) Avalanche Size:*** *Group-level comparison of average avalanche size (# of channels) for each patient.* ***(d) Inter-Avalanche Interval:*** *Group-level comparison of average inter-avalanche interval (ms) for each patient. Dashed lines indicate higher values in the OFF condition compared to ON, while solid lines represent higher values in the ON condition compared to OFF. For* ***a-d****, we tested the significance of the differences in the distributions between ON-levodopa and OFF-levodopa conditions using the paired t-test, resulting in the following effect sizes, measured as Cohen's d (denoted by d), and p-values (denoted by p): avalanche rate (d=0.64, p=0.058), duration (d=0.77, p=0.027), size (d=0.94, p=0.010), and inter-avalanche interval (d=1.04, p=0.006). The significance level are indicated as *** : p< 0.001, ** : p<0.01, *: p<0.05, ns = non-significant, p refers to p-value.*

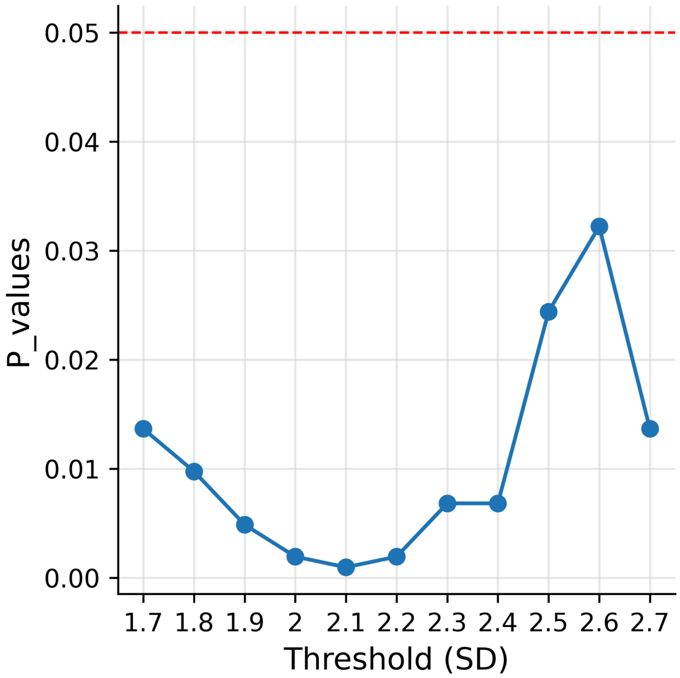

Supplementary Figure 15. Significance of average ATM differences with threshold changes*. Impact of threshold changes on the significance of ATM averages (across all edges) when comparing ON- and OFF-levodopa conditions. Statistical significance of the differences was tested using the Wilcoxon signed-rank test. The red dashed line represents the threshold p-value of 0.05.*
